# Cases in Practice

**Published:** 1858-06

**Authors:** L. H. Angell

**Affiliations:** Aurora, Ill.


ARTICLE III.

### CASE IN PRACTICE.

BY L. H. ANGELL, M. D., AURORA, ILL.

*February 4th.* Thursday evening, 8 o'clock 5 m., was called to see Mrs. K., and found symptoms as follows: face and hands of a scarlet color, dark appearance about the eyes, pupils contracted, conjunctiva slightly reddened, quick, feeble and rapid pulse, 130 at least; complains of great pain in back and through the whole abdomen, increased by placing hand over abdominal region; great thirst, calling for water continually; has vomited frequently; has taken nothing but "corn coffee and soda" to allay vomiting; says she is "unwell." Last had her menses "six or seven weeks since;" supposed she was pregnant; ex. per vaginam; some hæmorrhage; os and cervix swollen and painful to touch; os dilated sufficiently to admit index; says she has taken nothing to bring on menses. Introduced tampon in vagina, and gave morph. sul., gr. $\frac{1}{4}$, which was soon repeated. In half an hour she became more quiet, but says yet the pain is very severe; pulse became a little fallen. I left her for a short time, but was soon again summoned, and found her worse.

The hæmorrhage had not amounted to much, nor had she vomited, yet there was an aggravation of all the symptoms: pulse more rapid and feeble, countenance of a deeper scarlet, and lips purple; constant tossing about, and does not lie still one minute; most urgent thirst; extremities warm. Applied sinapisms to epigastrium, abdomen and back, also extremities, and gave a large dose of morphine. Sent for Doctor Howell. He came at a few minutes before eleven, and it being necessary for me to leave, he remained with the patient. There was no change for the better, but she continued to sink. She vomited some during the night, and there was occasional hæmorrhage; once she vomited a quantity of blood. She died at 7½ Friday A. M., eleven hours after I first saw her. Her husband says that he supposed abortion took place on Wednesday night; that on that evening she attended church, and did not retire until late; that she vomited in the night, and did not arise on Thursday morning until 9 A. M.; she then got up and partook of breakfast; none of the inmates of the house were disturbed by the sickness of Mrs. K. on Wednesday night. She had not complained of any symptom except pain in the back previously, as I can learn. She remained about the house on Thursday until 1 P. M., and then went to bed. She, prior to this, to appearance, had been a healthy woman, and had borne children. There were no cerebral symptoms or convulsions during her sickness.

AUTOPSY—present, Drs. Buck, Allaire, Howell, Hard, Gillet and Young—twenty-six hours after death.

External appearance. Skin of a dark scarlet blackness; cellular tissue of face and neck very much distended with gas. In dividing the integument over the sternum, a quantity escaped.

Abundance of adipose tissue, at least one and a half inches thick, at the line of section. Upon raising the sternum, the tissues beneath were seen to be of the same purple color as externally. Heart flaccid and entirely empty. A quantity of reddish fluid in pericardium. This resembled that found everywhere, which appeared to be blood in a fluid condition. No coagulum, or any evidence of fibrin, found anywhere. Liver greatly congested, and of a deep purple blackness; a portion of the right lobe tore easily upon raising it. The spleen also was more intensely congested than the liver, and its texture so softened that it tore apart on gently raising it. Stomach, externally, presented a deep redness, varying from a scarlet to a rose red, the greatest in the greater curvature. It contained a brownish tenacious mucus, and a quantity of gas. Internally, upon its posterior surface, a circular patch of the villous coat, some two inches in diameter, was elevated, resembling a number of bullæ clustered together. The whole villous surface was intensely congested, and at some points well-marked inflammation existed. There was a small number of dark-colored patches, bearing a resemblance to those produced by the application of the nitrate of silver to the skin. The intestines were of the same peculiar color, and at some points intensely congested, as was also the omentum. The uterus was of a dark purple color, intensely congested, its texture softened. Os enlarged and congested; internal surface covered with a dark tenacious mucus, and the appendages involved in the same peculiar congestion.

In view of the above peculiar pathological appearances, the evidences of gastric inflammation, the rapid disorganization of some of the abdominal viscera, the destruction of the fibrinous element of the blood, and the peculiar purple color of all the tissues, occurring in a subject previously healthy, having its commencement and termination within twenty-four hours, it was thought not unlikely that some substance of a highly-irritating property had been received into the stomach. In order to elucidate this point, and if possible make certain the pathology of the case, the stomach was shown to several medical gentlemen of Chicago, among whom I am informed were yourself and Prof. Blaney, who has now the specimen in his possession. If you, sir, or any one else, can, by the aid of these notes, together with such examination of the stomach as may be had without too much sacrifice, render more certain the etiology of this unfortunate case, you will confer a favor upon the profession and yours truly.

